# Higher prevalence of *NUDT15* rs116855232 compared to *TPMT* rs1142345 in a Chinese cohort and its implications for thiopurine therapy

**DOI:** 10.3389/fphar.2025.1660719

**Published:** 2025-09-18

**Authors:** Chenyu Zhao, Hui Huang

**Affiliations:** ^1^ Department of Gastroenterology, Henan Provincial People’s Hospital, People’s Hospital of Zhengzhou University, Zhengzhou, China; ^2^ Department of Medical Genetics, Hunan Province Clinical Research Center for Genetic Birth Defects and Rare Diseases, The Second Xiangya Hospital, Central South University, Changsha, China

**Keywords:** NUDT15, TPMT, thiopurine, gene polymorphism, Pharmacogenetics

## Abstract

**Background:**

Thiopurine drugs are widely used as immunosuppressants and chemotherapeutic agents in clinical practice, but their adverse effects significantly limit their clinical application. *TPMT* c.719A>G (rs1142345) and *NUDT15* c.415C>T (rs116855232) are the most common genetic polymorphisms influencing thiopurine drug toxicity, with notable differences in allele frequencies across diverse populations. However, there remains a paucity of research on the *NUDT15* c.415C>T polymorphism in the Chinese population.

**Methods:**

This study enrolled 571 Chinese patients. DNA samples were isolated, and polymerase chain reaction (PCR) was performed to amplify the *TPMT* c.719A>G and *NUDT15* c.415C>T in each sample. PCR products were genotyped via Sanger sequencing to identify the allelic frequencies of these polymorphisms. Additionally, we compared the detection rate of *NUDT15* c.415C>T and *TPMT* c.719A>G for thiopurine drug toxicity in the cohort.

**Results:**

The minor allele frequencies of *NUDT15* c.415C>T and *TPMT* c.719A>G were determined to be 12.52% and 2.36%, respectively. The detection rate of the *NUDT15* c.415C>T polymorphism was significantly higher than that of *TPMT* c.719A>G (23.47% vs. 4.55%, P < 0.001).

**Conclusion:**

*NUDT15* c.415C>T yielded a higher carrier rate than *TPMT* c.719A>G in this cohort. And broader panels could shift absolute yields. These findings highlight the critical role of *NUDT15* c.415C>T genotyping in guiding precision therapy with thiopurine drugs.

## Introduction

Thiopurine drugs, including azathioprine, mercaptopurine, and thioguanine, are widely employed as immunosuppressants and chemotherapeutic agents. In immunosuppressive therapy, they are used to treat inflammatory bowel disease, myasthenia gravis, rheumatoid arthritis, and to prevent organ transplant rejection. As chemotherapeutics, they play a key role in managing acute leukemia and chronic myeloid leukemia. Despite their clinical utility, their use is constrained by severe, potentially life-threatening adverse drug reactions (ADRs), such as myelosuppression, alopecia, hepatotoxicity, and pancreatitis (Marinaki and Arenas-Hernandez, 2020). These toxicities are strongly linked to genetic polymorphisms in two key enzymes: thiopurine S-methyltransferase (TPMT) and nucleoside diphosphate-linked moiety X-type motif 15 (NUDT15) (Relling et al., 2019).

TPMT is primarily responsible for inactivating thiopurine drugs. Reduced or absent TPMT activity leads to elevated levels of thiopurine active metabolites, thereby increasing the risk of toxicity. Genetic polymorphisms in the *TPMT* gene significantly influence enzyme activity. In Western populations, the *TPMT* variants ∗3A and ∗3C account for 90% of cases with low enzyme activity and are associated with leukopenia (Meng et al., 2018). *TPMT*∗3A is composed of two single nucleotide polymorphisms (SNPs): rs1800460 (c.460G>A) and rs1142345 (c.719A>G). Unlike *TPMT*∗3A, which is defined by a combination of two SNPs, *TPMT*∗3C is composed solely of the single SNP variant rs1142345. In contrast to populations of European descent, loss-of-function *TPMT* alleles exhibit lower prevalence in East Asian populations. In Chinese cohorts, the c.719A>G (rs1142345) allele frequency typically ranges from ∼1 to 3%, with TPMT*3C (c.719A>G) as the predominant star allele (Mao et al., 2021; Zhou and Lauschke, 2022). By comparison, carrier prevalence of *TPMT* loss-of-function alleles in European populations is approximately 10% (Teml et al., 2009). Notably, the low prevalence of *TPMT* variants in East Asians suggests that alternative genetic factors contribute to the higher incidence of thiopurine-related ADRs in this population (Liang et al., 2016).

NUDT15 negatively regulates thiopurine activation, and loss-of-function variants lead to accumulation of cytotoxic metabolites (Man et al., 2019). The Clinical Pharmacogenetics Implementation Consortium (CPIC) included nine *NUDT15* single-nucleotide polymorphisms in its 2018 dosing guidelines for thiopurines (Relling et al., 2019). The most frequent variant is *NUDT15∗3* (rs116855232, c.415C>T) (Mao et al., 2021). Additionally, minor allele frequency (MAF) of *NUDT15∗2* in East Asians is about 3%. It is important to note that NUDT15*2 is defined by two specific SNPs: rs116855232 (c.415C>T) and rs746071566 (c. 55_56insGAGTCG). This variant has been strongly associated with thiopurine toxicity (Yang et al., 2014; Zhang et al., 2018). It exhibits a higher prevalence in Asian populations compared to European or African populations (Yang et al., 2015; Khaeso et al., 2021). Unlike *TPMT*, *NUDT15* variants show a distinct ethnic distribution, underscoring their critical role in predicting ADRs in East Asians.

In addition to *TPMT* and *NUDT15*, other genes involved in the thiopurine drugs metabolic pathway have been explored for their association with treatment-related adverse effects. For example, genetic variants at the *ITPA* locus (e.g., rs1127354 and rs7270101) are associated with reduced enzymatic activity, which may elevate toxicity risk through the accumulation of the potentially harmful metabolite thioinosine triphosphate (Moradveisi et al., 2019; Ali et al., 2023). Given their status as the most prevalent genetic variants implicated in thiopurine metabolism, *TPMT* c.719A>G (rs1142345) and *NUDT15* c.415C>T (rs116855232) were selected for investigation in this study.

The frequencies of *NUDT15* and *TPMT* variants exhibit significant ethnic diversity. Notably, there is a paucity of research on the *NUDT15* c.415C>T polymorphism among the Chinese population, particularly the Han ethnic group. The objectives of the study were twofold: (1) to determine the allelic frequencies of these two variants in a Chinese cohort (predominantly Han ethnicity); and (2) to evaluate their comparative utility in predicting thiopurine drug toxicity, with the aim of optimizing healthcare resource allocation in China, a developing nation with a large population.

## Materials and methods

### Study participants and data collection

This retrospective clinical study included 571 patients who underwent *TPMT* c.719A>G and *NUDT15* c.415C>T genotyping at the Second Xiangya Hospital. Data extracted from electronic medical records comprised patients’ age, gender, nationality, diagnosis, pharmacogenetic testing results, and the department of the ordering physicians. The study was approved by the Ethics Committee of the Second Xiangya Hospital, Central South University (approval number: 141225S046).

### Pharmacogenetics testing

Genomic DNA was extracted from peripheral blood samples. We performed genotyping for *TPMT* c.719A>G (rs1142345) and *NUDT15* c.415C>T (rs116855232) using PCR-Sanger sequencing. Genotyping results were documented and returned in standardized report formats within the electronic medical record system. Notably, our assay did not include interrogation of *TPMT* c.460G>A (rs1800460) or *NUDT15* c.55_56insGAGTCG (rs746071566). Consequently, this methodological limitation precludes definitive assignment of TPMT*3A versus *3C alleles (or detection of *3B) and hinders discrimination between NUDT15*2 and *3 alleles. Therefore, all results are reported as variant-specific frequencies rather than star-allele frequencies, with interpretations explicitly contextualized within the scope of our single-locus assay. Additional clinically relevant alleles recommended by CPIC/Association for Molecular Pathology (AMP) were not interrogated and fall outside the current assay’s design.

### Allele frequencies and sensitivity analysis

Allele frequencies of *TPMT* c.719A>G and *NUDT15* c.415C>T were calculated as: Allele frequency = [(Numbers of heterozygotes + Numbers of homozygotes × 2)/Total sample numbers × 2] × 100%.

In this study, each patient underwent both genotyping tests simultaneously. Carriage of either the *TPMT* c.719A>G or *NUDT15* c.415C>T was defined as a positive result, as individuals with intermediate or poor metabolizer phenotypes require adjustment of thiopurine drug dosages. We compared the sensitivity of these two variants for guiding thiopurine dosing decisions in the Chinese (East Asian population).

### Statistical analysis

Data were analyzed with SPSS (version 20; SPSS and SAS, version 9.2; SAS Institute, IBM Corp., Armonk, NY). Statistical tests of significance were conducted by paired Chi-square test using McNemar’s test. The criterion for statistical significance was p < 0.05.

## Results

### Characteristics of study participants

This study included 343 females and 228 males, with a median age of 43 years (range: 5–84 years). The vast majority were of Han Chinese ethnicity (99.12%, 566/571). Other ethnic groups included Tujia (0.35%, 2/571), Miao (0.35%, 2/571), and Dong (0.18%, 1/571). This distribution was largely consistent with the 2020 National Population Census of China, which reported Han Chinese as the majority (91.1%). Patients were primarily from the Departments of Neurology (52%, 297/571), Rheumatology (22.9%, 131/571), and Gastroenterology (22.6%, 129/571). Most diagnoses were non-malignant, including myasthenia gravis, autoimmune myositis, and Crohn’s disease. Patient demographic and clinical characteristics are summarized in Table 1 and Supplementary Material.

**TABLE 1:** Characteristics of the patients in our cohort (n = 571).

Characteristics	
Age, years	
Median	43
Range	5–84
Gender	
Female	343 (60.1%)
Male	228 (39.9%)
Nationality	
Ethnic Han	566 (99.1%)
Other ethnic groups	5 (0.9%)
Clinical departments	
Department of neurology	297 (52%)
Department of rheumatology	131 (22.9%)
Department of gastroenterology	129 (22.6%)
Others	14 (2.5%)

Others: Departments of dermatology, infectious diseases, geriatrics, respiratory and ophthalmology.

### Frequency of *TPMT* c.719A>G and *NUDT15* c.415C>T variants

Among all 571 patients, 9 were homozygous for *NUDT15* c.415C>T (TT), 125 were heterozygous (TC), and 437 were wild-type (CC). Conversely, only 1 patient was homozygous for *TPMT* c.719A>G (GG), 25 were heterozygous (AG), and 545 were wild-type (AA). The MAFs were 12.52% for *NUDT15* c.415C>T and 2.36% for *TPMT* c.719A>G, respectively (Table 2 and Supplementary Material).

**TABLE 2:** Distribution of *TPMT* c.719A>G and *NUDT15* c.415C>T.

Genotype	Number of patients			Frequency (%)
	WT	HET	HOM	
*TPMT* c.719A>G	545	25	1	2.36
*NUDT15* c.415C>T	437	125	9	12.52

WT:wild type (for the allele of interest); HET: heterozygote; HOM: homozygous for the variant allele.

### Comparison for detection rate between two variants

The positive detection rates for *TPMT* c.719A>G and *NUDT15* c.415C>T were 4.55% (26/571) and 23.47% (134/571), respectively. A paired Chi-square test revealed a statistically significant difference positive rates between *NUDT15* c.415C>T and *TPMT* c.719A>G (Table 3, P < 0.001). These results indicate that *NUDT15* c.415C>T genotyping is more sensitive than *TPMT* c.719A>G testing in the Chinese population. Additionally, seven patients carried heterozygous mutations in both *NUDT15* c.415C>T (TC) and *TPMT* c.719A>G (AG).

**TABLE 3:** Frequency distribution/contingency table for preparation of the chi-squared test (resulting statistics are presented in the text).

	*TPMT* c.719A>G			Total
		+	-	
*NUDT15* c.415C>T	**+**	7	127	134
	**-**	19	418	437
Total		26	545	571

A total of 153 patients (26.8%, 153/571) were predicted to have intermediate or poor TPMT/NUDT15 activity. While *NUDT15* c.415C>T testing adds 22.25% to the diagnostic yield of *TPMT* c.719A>G alone, *TPMT* c.719A>G complements *NUDT15* c.415C>T by identifying an additional 3.33% of at-risk patients negative for *NUDT15* c.415C>T variants. These patients were recommended to adjust thiopurine dosages or switch to alternative therapies. In this cohort, no serious adverse events were observed except in two cases. Both patients had Crohn’s disease. In our hospital, clinicians recommend conducting *TPMT* c.719A>G and *NUDT15* c.415C>T genetic testing prior to initiating thiopurine therapy. And according to CPIC guidelines, intermediate metabolizers should initiate thiopurine therapy with reduced starting doses (30%–80% of normal dose). Poor metabolizers should consider alternative non-thiopurine immunosuppressant therapy in non-malignant conditions. Unfortunately, the two patients initially refused to undergo genetic testing and received conventional thiopurine dosages. Subsequently, they developed agranulocytosis. Pharmacogenetic testing performed afterward revealed they were homozygous for *NUDT15* c.415C>T (Table 4).

**TABLE 4:** Characteristics of the 2 patients who developed agranulocytosis.

Clinical Information of Patients	Patient 1	Patient 2
Age (years)	31	43
Gender	Male	Male
Nationality	Han	Han
Diagnosis	Chron’s disease	Chron’s disease
*NUDT15* c.415C>T genotype	HOM	HOM
*TPMT* c.719A>G genotype	WT	WT
WBC	0.4*10^9^/L	0.98*10^9^/L
RBC	3.45*10^12^/L	4.47*10^12^/L
PLT	88*10^9^/L	69*10^9^/L
NEUT	0.05*10^9^/L	0.1*10^9^/L

WBC: white blood cells, RBC: red blood cell, PLT: platelets, NEUT: neutrophil coun.

## Discussion

Previous studies have investigated the association between thiopurine drug-induced ADRs and genes including *TPMT, ITPA, NUDT15, GST, MRP4, HGPRT, IMPDH,* and *XO*. Among these, *TPMT, NUDT15*, and *ITPA* are the most extensively studied (Chen et al., 2021; Suzuki et al., 2023; Salazar et al., 2024). Notably, the U.S. Food and Drug Administration (FDA) recommends determining patient *TPMT* genotypes prior to drug administration, while the CPIC guidelines additionally advise assessing *NUDT15* genotypes before initiating thiopurine therapy (Relling et al., 2019). Therefore, this study was intentionally designed to focus on *NUDT15* and *TPMT*, given their well-established role as primary contributors to thiopurine toxicity in the study cohort, although the *ITPA* gene has been implicated in thiopurine-induced ADRs.

**FIGURE 1:**
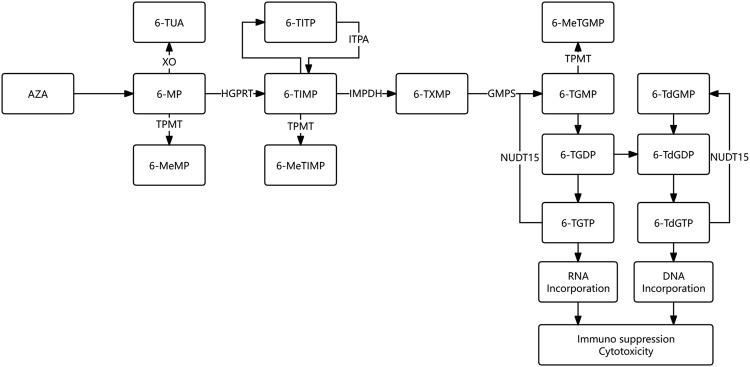
Thiopurine metabolism pathway XO: Xanthine oxidase; TPMT: Thiopurine S-methyltransferase; HGPRT: Hypoxanthine guanine phosphoribosyl transferase; ITPA: Inosine triphosphate pyrophosphatase; IMPDH: Inosine monophosphate dehydrogenase; GMPS: Guanosine monophosphate synthetase; NUDT15: Nucleoside diphosphate-linked moiety X-type motif 15; AZA: Azathioprine; 6-MP:6-Mercaptopurine; 6-TUA: 6-Thiouric acid; 6-MeMP: 6-Methylmercaptopurine; 6-TIMP: 6-Thioinosine monophosphate; 6-TITP: 6-Thioinosine triphosphate; 6-MeTIMP: 6-Methylthionosine monophosphate; 6-TXMP: 6-Thixanthosine 5′-monophosphate; 6-TGMP: 6-Thioguanosine monophosphate; 6-TGDP: 6-Thioguanosine diphosphate; 6-TGTP: 6-Thioguanosine triphosphate; 6-MeTGMP: 6-Methylthioguanine monophosphate; 6-TdGMP: 6-thio-deoxyguanosine monophosphate; 6-TGDP: 6-thio-guanosine diphosphate; 6-TdGTP: 6-thio-deoxyguanosine triphosphate. This figure is adapted from previously published works (Tanaka and Saito, 2021; Suzuki et al., 2023).

Azathioprine (AZA) acts as a prodrug of 6-mercaptopurine (6-MP) and undergoes nonenzymatic conversion to 6-MP within erythrocytes. Subsequently, 6-MP is metabolized into various derivatives by three key enzymes: xanthine oxidase (XO), TPMT, and hypoxanthine guanine phosphoribosyl transferase (HGPRT). The thiopurine metabolism pathway is depicted in Figure 1 (Matsuoka, 2020; Tanaka and Saito, 2021; Suzuki et al., 2023). Reduced TPMT activity leads to accumulation of 6-thioguanine nucleotides (6-TGN). Decreased NUDT15 activity increases thiopurine triphosphate levels. Inosine triphosphate pyrophosphatase (ITPA) converts 6-thioinosine triphosphate (6-TITP) to 6-thioinosine monophosphate (6-TIMP), and diminished ITPA activity is hypothesized to cause 6-TITP accumulation. All of these conditions could result in an increased risk of adverse drug reactions.

To date, over 40 *TPMT* alleles (*TPMT*∗2–∗41) have been identified in individuals with TPMT deficiency (Iu et al., 2017). The frequency of *TPMT* genetic polymorphisms varies significantly across ethnic groups, with an approximate prevalence of 3% in Asians—substantially lower than that in European populations (Fangbin et al., 2012; Matsuoka, 2020). The most common polymorphism is *TPMT* c.719A>G, which exhibited an overall prevalence of 2.36% in our Chinese cohort (predominantly Han ethnicity). The TPMT enzyme is central to thiopurine metabolism, and the *TPMT*∗3C polymorphism represents a risk factor for thiopurine intolerance (Cardoso de Carvalho et al., 2020). This variant induces protein instability and impairs TPMT enzymatic activity (Evans, 2004), leading to accumulation of thiopurine nucleoside active metabolites and subsequent cytotoxicity. Conversely, the NUDT15 enzyme dephosphorylates thiopurine triphosphate—the active metabolite incorporated into DNA—into its monophosphate form (Tanaka and Saito, 2021). *NUDT15*∗3 is recognized as a loss-of-function variant (Moriyama et al., 2016), causing elevated thiopurine triphosphate levels and exacerbating thiopurine-induced cytotoxic effects, including myelosuppression and alopecia. In our cohort, *NUDT15* c.415C>T had an overall prevalence of 12.52% in the Chinese population (predominantly Han ethnicity).

During thiopurine treatment, the incidence of leukopenia ranges from 15% to 40% in Asian populations (Takatsu et al., 2009; Kim et al., 2010; Qiu et al., 2015), significantly higher than the approximately 3% reported in Western populations (Lewis et al., 2009; Sood et al., 2015). Severe leukopenia affects approximately 1% of Asian patients (Asada et al., 2016), underscoring the critical need for pharmacogenetic testing in these populations. As a developing East Asian nation with a population exceeding 1.4 billion, China faces regional disparities in economic development and limited public healthcare funding. From a health economics perspective, there is a pressing need to deliver cost-effective personalized medication guidance to patients within constrained financial resources.

In our cohort, the prevalence of *TPMT* c.719A>G was significantly lower than that of *NUDT15* c.415C>T (2.36% vs. 12.52%). The positive detection rates for *TPMT* c.719A>G, *NUDT15* c.415C>T, and both indexes testing were 4.55%, 23.47%, and 26.8%, respectively. Compared with testing *NUDT15* c.415C>T alone, simultaneous testing of both polymorphisms only increased the positive rate by 3.33%. Additionally, only one patient was homozygous for *TPMT* c.719A>G. These findings suggest that single-locus testing for *NUDT15* c.415C>T may be a clinically acceptable strategy in China, although the CPIC guideline for thiopurine dosing (Relling et al., 2019) still recommends comprehensive detection of all relevant polymorphisms.

The present study is subject to several limitations. The current study focused on *NUDT15* c.415C>T and *TPMT* c.719A>G variants, but did not include the complete allele panel recommended by CPIC/AMP guidelines. This may lead to underreporting clinically relevant alleles and does not fully align with standardized testing protocols. It prioritized *TPMT* c.719A>G and *NUDT15* c.415C>T due to their established clinical relevance in East Asian populations, where they represent the most frequently observed variants associated with thiopurine-induced toxicity (Relling et al., 2019; Mao et al., 2021). This may have resulted in incomplete characterization of genetic contributions to ADRs of thiopurine drugs. Due to the two-site design of our genotyping assay, inference of star alleles, which relies on haplotypic combinations of multiple variants, was not feasible in this study. Star-allele inference for *TPMT* and *NUDT15* necessitates multi-locus haplotype analysis (e.g., TPMT*3A = c.460G>A+ c.719A>G; NUDT15*2 = c.415C>T + c.55_56insGAGTCG). Owing to our assay’s exclusive interrogation of c.719A>G and c.415C>T, we may have overestimated the prevalence of TPMT*3C (as some carriers could harbor the *3A haplotype) and NUDT15*3 (as some carriers may actually carry the *2 allele). Future investigations should incorporate all AMP/CPIC Tier-1 variants to enable accurate star-allele assignment and robust haplotype-based genotyping. Besides, the lack of metabolite data represents a limitation of the current study. Future investigations incorporating simultaneous measurement of thiopurine metabolites and genetic variants will help elucidate how allele status affects drug metabolism and, ultimately, clinical responses.

## Conclusion

In summary, this study revealed that *NUDT15* c.415C>T yielded a higher carrier rate than *TPMT* c.719A>G in this cohort (predominantly Chinese Han ethnicity). Compared with *TPMT* c.719A>G, *NUDT15* c.415C>T demonstrated greater suitability for predicting thiopurine drug toxicity in Chinese patients. It is important to note that this study only interrogated two single variants. Broader genetic panels may alter the absolute carrier yields. These findings highlight the critical role of *NUDT15* c.415C>T genotyping in optimizing precision therapy for thiopurine-based treatments.

## Data Availability

The original contributions presented in the study are included in the article/supplementary materials.
